# Idiosyncratic genome evolution of the thermophilic cyanobacterium *Synechococcus* at the limits of phototrophy

**DOI:** 10.1093/ismejo/wrae184

**Published:** 2024-09-25

**Authors:** C Logan Pierpont, Jacob J Baroch, Matthew J Church, Scott R Miller

**Affiliations:** Division of Biological Sciences, The University of Montana, 32 Campus Dr. #4824, Missoula, MT 59812, United States; Division of Biological Sciences, The University of Montana, 32 Campus Dr. #4824, Missoula, MT 59812, United States; Division of Biological Sciences, The University of Montana, 32 Campus Dr. #4824, Missoula, MT 59812, United States; Division of Biological Sciences, The University of Montana, 32 Campus Dr. #4824, Missoula, MT 59812, United States

**Keywords:** thermophile, adaptation, cyanobacteria, genomes, hot springs, horizontal gene transfer

## Abstract

Thermophilic microorganisms are expected to have smaller cells and genomes compared with mesophiles, a higher proportion of horizontally acquired genes, and distinct nucleotide and amino acid composition signatures. Here, we took an integrative approach to investigate these apparent correlates of thermophily for *Synechococcus* A/B cyanobacteria, which include the most heat-tolerant phototrophs on the planet. Phylogenomics confirmed a unique origin of different thermotolerance ecotypes, with low levels of continued gene flow between ecologically divergent but overlapping populations, which has shaped the distribution of phenotypic traits along these geothermal gradients. More thermotolerant strains do have smaller genomes, but genome reduction is associated with a decrease in community richness and metabolic diversity, rather than with cell size. Horizontal gene transfer played only a limited role during *Synechococcus* evolution, but, the most thermotolerant strains have acquired a *Thermus* tRNA modification enzyme that may stabilize translation at high temperatures. Although nucleotide base composition was not associated with thermotolerance, we found a general replacement of aspartate with glutamate, as well as a dramatic remodeling of amino acid composition at the highest temperatures that substantially differed from previous predictions. We conclude that *Synechococcus* A/B genome diversification largely does not conform to the standard view of temperature adaptation. In addition, carbon fixation was more thermolabile than photosynthetic oxygen evolution for the most thermotolerant strains compared with less tolerant lineages. This suggests that increased flow of reducing power generated during the light reactions to an electron sink(s) beyond carbon dioxide has emerged during temperature adaptation of these bacteria.

## Introduction

Temperature has pervasive effects at all levels of biological organization [1, 2]. Revealing both the mechanisms of and constraints on adaptation to temperature extremes has far-reaching implications for our understanding of the origins of biological diversity, how organisms respond to a changing environment and the nature of Life’s metabolic limits [3, 4]. Investigations of the genomes of thermophilic microorganisms have identified several apparent correlates of thermophily that shed light on these mechanisms. Thermophiles have been reported to have smaller genomes than their less thermotolerant relatives, potentially due to selection for small cell size to reduce maintenance costs [5, 6]. Further, they have been proposed to have more stable informational macromolecules through increased GC content of structural RNAs [7–9] as well as higher purine levels [10–12]. Moreover, although proteins may be stabilized by diverse molecular mechanisms [13–19], strong general correlates between organismal amino acid composition and optimal growth temperature (T*_opt_*) have been identified; this includes the increased use of charged and bulky hydrophobic residues [20], with the abundance of seven amino acids (IVYWREL) being the strongest predictor of thermotolerance [21]. These changes may enhance stability through a greater number of ionic bonds and increased packing density within the protein core, respectively [22]. Finally, horizontal gene transfer (HGT) is thought to have been essential to the evolution of thermophily in bacteria [23], with up to a quarter of genes transferred from hyperthermophilic archaea [24, 25].

Members of the *Synechococcus* A/B (*Syn*AB) group (also called *Thermostichus*) form an early branching clade of cyanobacteria [26] that includes the most heat-tolerant phototrophs on Earth [27]. Endemic to alkaline geothermal environments in western North America at temperatures between ~50 and 73°C [27], *Syn*AB vary extensively in thermal performance, the most thermotolerant of which are the only cyanobacteria capable of growth above 65°C [28–30]. Previous *Syn*AB phylogenies have been marked by limited taxon sampling and/or reliance on single genes [29, 31, 32]. Consequently, it has remained unresolved whether divergent *Syn*AB lineages have a unique, ancient origin with subsequent dispersal, or, alternatively, whether there have been independent adaptive radiations within distinct geographic regions. Further, although the most thermotolerant *Syn*AB have evolved enhanced thermostability of the carbon-fixing enzyme RuBisCO, oxygen-evolving photosystem II, and the light-harvesting phycobilisome [33, 34], the general genomic mechanisms underlying the evolution of thermotolerance are still generally poorly understood. Here, we combine evolutionary genomics and physiology for a large sample of laboratory strains isolated from Yellowstone NP and Oregon to investigate temperature adaptation during *Syn*AB diversification. Our results challenge the conventional paradigm for the evolution of thermophily.

## Materials and methods

### Collection of mat samples

We collected samples from alkaline geothermal environments in the Midway and Lower Geyser Basins of Yellowstone National Park, WY (June and September 2018, July 2019) and, in August 2018, from Hunter’s Hot Springs, OR (Supplementary Table 1). For all collections, we used a sterile syringe to transfer ~10 ml of microbial mat to a sterile 15 ml conical centrifuge tube and also recorded the in situ temperature (Supplementary Table 1). We stored samples at ambient temperature in the dark until processed, no later than 48 h after collection.

### Isolation and growth of *Syn*AB strains

Unless otherwise specified, we cultured cells in mineral salts medium D [35], designed to mimic water from Hunter’s Hot Springs, OR. We homogenized samples via vortex and then estimated *Synechococcus* cell densities with hemocytometer counts. To isolate individual colonies on a solid substrate, we diluted vacuum filtered samples onto a sterile 0.2 μm glass fiber filter, which was then submerged in 75 ml medium. All flasks were incubated in a growth chamber at or near their source collection temperature with ~75 μmol photons m^−2^ s^−1^ cool white fluorescent light and a 12 h/12 h photoperiod. After 1–2 weeks, we sterilely removed filters containing cyanobacterial colonies and sterilely transferred isolated colonies to flasks containing fresh medium to grow for an additional 3 weeks. We sequentially repeated this dilution-filtration approach at least twice and selected putative clones for further analysis. To confirm the presence of *Syn*AB, we extracted DNA using BioRad’s InstaGene Matrix protocol and next amplified a fragment of the cyanobacterial 16S rRNA gene by PCR using primers CYA359F and PLG2.3R, as previously described [29]. Cycling conditions were 50 cycles of 95°C for 30 s, 55°C for 30 s, and 72°C for 30 s. We purified amplicons with the Zymo Research DNA Clean & Concentrator Kit, followed by Sanger sequencing (GENEWIZ, Azenta Life Sciences). In addition, we grew outgroup strain *Synechococcus* Nb3U1 [36] at 45°C in liquid medium BG11 buffered with 10 mM HEPES (pH 8.0).

### Genome sequencing, assembly, and annotation

We extracted genomic DNA from cells using Qiagen’s DNeasy PowerBiofilm Kit according to manufacturer protocols. We prepared sample libraries for paired-end, short-read sequencing with a Nextera DNA flex kit, which was followed by 150 cycles of sequencing on a NextSeq 550 platform (Illumina). We checked read quality using FastQC v0.11.9 (Babraham Bioinformatics) and removed Illumina adapter sequences with Trimmomatic v0.36 [37].

We de novo assembled draft genomes with SPAdes v3.12.0 [38] and then removed contigs shorter than 1 kb. Because these cultures were not axenic, we designed a refinement pipeline using Kraken v2.1.2 [39] and BLAST+ v2.2.31 [40] to identify cyanobacterial contigs, as previously described [41]. We generated coverage histograms to identify outlier contigs and used Bandage v0.8.1 [42] to remove these outlier contigs and to estimate the mean coverage of the assembly. We estimated genome statistics using QUAST v4.5 [43] and completeness of each assembly by BUSCO v5.2.2 [44].

In addition, we selected six representative strains from different major *Syn*AB clades along with outgroup strain NB3U1 for long-read sequencing: W60.1 (collection temperature = 61.3°C); H55.10 (55.6°C), H60.4 (62°C); W60.3 (64.8°C); H70.2 (67.5°C); and W70.1 (71.7°C). Selection was based on genome quality of the short-read assemblies, indicated by N50 metrics and BUSCO analyses. We extracted high molecular weight genomic DNA using Qiagen’s Genomic-tip 20/G protocol, prepared sequencing libraries using Nanopore’s Ligation Sequencing kit, and then sequenced samples for 48 h with a Nanopore MinION sequencer using a FLO-MIN106D flow cell with R9.4.1 chemistry. We called bases with GUPPY v4.5.4 [45] generated and refined hybrid genome assemblies using SPAdes v3.12.0, and annotated with NCBI’s Prokaryotic Genome Annotation Pipeline [46], available at NCBI BioProject PRJNA795194.

### Phylogenomics

We identified single-copy orthologous sequences with OrthoFinder v2.5.4 [47]. These were individually aligned using MUSCLE v5.1 [48], then concatenated using MEGAX v10.2.6 [49]. We constructed a maximum likelihood phylogeny with IQtree v2.0.6 [50] for 118 301 aligned amino acid sites using the JTT + F + R3 model of sequence evolution, identified as the best model by AIC and BIC with ModelFinder [51]. The tree was outgroup rooted with strain Nb3U1 and ultrafast bootstrap replicated with 1000 replicates. To estimate the degree of congruence between the concatenated species tree and individual gene trees, we also reconstructed trees for each ortholog by maximum likelihood and then estimated the gene concordance factor (gCF; [52]); in addition, we used RAxML v8.2.10 [53] to estimate internode certainty (IC; [54]).

### Comparative genomics

We used ROARY v3.12.0 [55] to identify single-copy orthologs shared within *Syn*AB clades, respectively. We used the default 95% amino acid identity BLASTp cutoff in all cases, with the exception of Clade I sequences, for which a 90% cutoff was used for its broader range of sampled diversity. To identify both the *Syn*AB core genome and genes unique to each clade, we next used ROARY to compare clade core genomes. Here, we used an empirically determined 70% amino acid identity cutoff based on the results of a local tBLASTx search between the Clade I and Clade VI cores. Putatively unique genes within a clade were further subjected to local BLASTx searches against the individual amino acid annotation files of all sampled *Syn*AB genomes with significance thresholds of ≥50% query coverage and an E-value ≤1e^−50^. Because the E-value metric is dependent on both subject database size and query length [40], in cases where no significant hits were found, the cutoff was relaxed to either half the E-value of the most significant within-clade hit or 1e^−10^, whichever was lower. Average nucleotide identities of genomes were estimated with the OrthoANIu algorithm [56].

Genome-wide GC content, GC content at the three codon positions of protein-coding genes, % non-coding DNA, and amino acid composition were determined using custom Python scripts. EggNOG-mapper v2.0 [57] and KEGG pathway reconstruction [58] were used to identify metabolic modules. For models investigating the relationships among traits and environmental temperature, we took a phylogenetic generalized least squares approach using the “ape” and “nlme” R packages. The variance–covariance matrix of the error term in each model was obtained with ape from branch-length data for the *Syn*AB phylogeny using the “corMartins” correlation structure; for all models, the phylogenetic correlation had been erased (i.e. observations were statistically independent).

### Growth experiments

We determined the temperature dependence of growth rate for a representative *Syn*AB strain from each of the six major clades, as well as for outgroup strain NB3U1. Starting at a strain’s maintenance temperature, we inoculated triplicate flasks containing 75 ml of medium with growing cells to a final OD_750_ of ~0.005, determined with a Beckman Coulter DU 530 spectrophotometer (Indianapolis, IN). Cells were grown under ~100 μmol photons m^−2^ s^−1^ of cool white fluorescent light with a 12 h/12 h photoperiod. We measured OD_750_ every 48 h using 2 ml of homogenized culture for at least three generations of growth, then estimated generation times from the exponential growth phase. This procedure was repeated at ~5°C increments above and below the starting temperature until no growth was observed. We used cells from a randomly selected replicate to inoculate flasks for the following treatment.

### Carbon fixation assays

We diluted growing cells cultured under normal maintenance conditions to an OD_750_ of 0.05 in 410 ml medium D. To a 70 ml aliquot of this cell suspension, we added H^14^CO_3_ to a final concentration of 3700 Bq ml ^−1^ and gently swirled to mix. To determine the specific activity, we subsampled a 250 μL aliquot of radiolabeled culture and delivered it to a vial containing 500 μL of β-phenylethylamine. Thirteen 5 ml aliquots were dispensed into 20 ml scintillation vials, which were then placed in a photosynthetron plumbed to a temperature controlled water bath such that each vial was exposed to a unique irradiance (μmol photons m^−2^ s^−1^): 0, 16, 32, 105, 150, 216, 306, 364, 462, 715, 957, 1288, and 1504. Vials were incubated for 1.5 h, then vacuum filtered onto a 0.2 μm Millipore MF cellulose acetate filter. This procedure was repeated at four different temperatures for each strain: 45, 55, 60, and 70°C. We next transferred each filter to a new 20 ml scintillation vial and added 1 ml 1 N HCl to remove unassimilated H^14^CO_3_; vials were passively vented overnight in a fume hood. Ten ml scintillation cocktail (Ultima Gold) was then added to each vial, capped tightly, vortexed, and radioactivity was quantified (as disintegrations per minute) on a liquid scintillation counter (Tri-Carb 3110TR). We calculated carbon assimilation from the ratio of the radioactivity of the samples relative to the specific activity total inorganic carbon available in the medium. We determined chlorophyll *a* concentrations by filtering 5 ml of each culture onto 0.45 μm cellulose acetate filters; filters were placed in 7 ml borosilicate tubes containing 5 ml of 90% acetone for 3 days at −20°C, after which extracts were analyzed fluorometrically. We normalized rates of carbon assimilation to chlorophyll *a* concentrations and fit the data with the Platt photosynthesis versus irradiance model [59] by hierarchical Bayesian Markov chain Monte Carlo with the RStan package.

### Oxygen evolution assays

We gently homogenized aliquots of growing cells using a flame-sterilized, glass Dounce homogenizer, pelleted (5500 × g for 30 min), and resuspended in D medium to 1.0 × 10^7^ cells ml ^−1^. Cells were incubated in a cuvette immersed in a temperature-controlled water bath. Samples were dark-acclimated for 10 min, then exposed to a rapid pulse of saturating light, followed by sequential 1-min exposures to a range of light intensities (1, 2, 8, 36, 55, 86, 125, and 219 μmol photons m^−2^ s^−1^) with a MINI-PAM fluorometer (Heinz Walz GmbH). We measured cuvette oxygen concentration at 1 s intervals with an ultra-fast responding OX-100 microsensor (Unisense), calibrated according to manufacturer instructions, and estimated rate of oxygen evolution (μmol O_2_ L^−1^ s^−1^) from the slope over 5–10 s. This process was repeated at 45, 55, and 60°C.

### Cell size measurements

For representative strains spanning the range of *Syn*AB thermotolerance, we prepared wet mounts of growing cells cultured at their thermal optimum. Slides were viewed with a compound light microscope with an attached camera. Using the same camera settings and magnification for all images, we took three images along with a micrometer scale. Using the micrometer scale to calibrate ImageJ (https://imagej.net/ij/index), we measured dimensions of 10 randomly selected cells from each of the three images.

## Results and discussion

### Evolutionary origins of the most extreme phototrophs on earth

**Figure 1:**
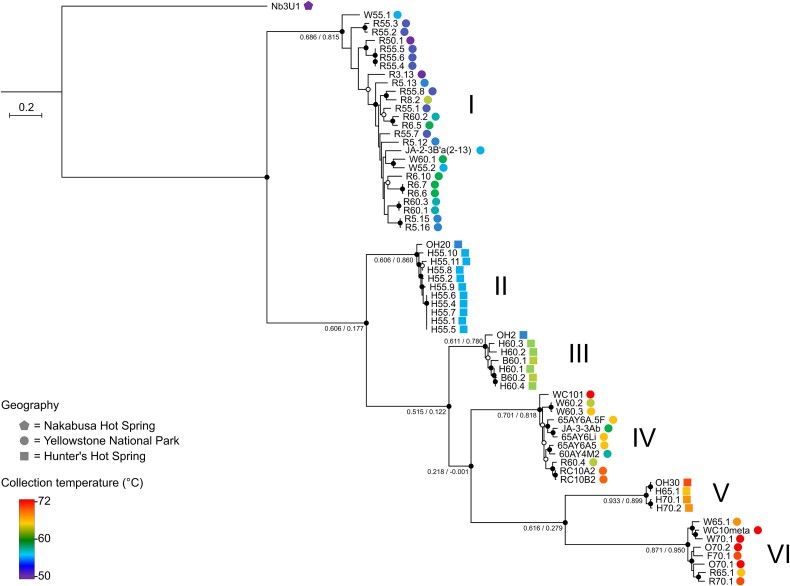
Genome-wide maximum likelihood *Syn*AB phylogeny reconstructed from a concatenated alignment of 118 301 amino acid sites derived from 404 single-copy orthologous gene sequences. The tree was outgroup-rooted with *Synechococcus* strain Nb3U1. Bootstrap values of 100% are indicated by closed circles, and values greater than 95% are indicated by open circles. Numbers at nodes indicate gCF and internode certainty values, respectively. Tree scale is in units of amino acid substitutions per site.

We reconstructed a genome-wide maximum likelihood phylogeny (Fig. 1) for 66 *Syn*AB laboratory strains (Supplementary Table 1) isolated from along the thermal gradients of hot springs in Yellowstone National Park, WY (YNP) and Oregon (OR). Nearly all of our draft genome assemblies were essentially complete based on comparable BUSCO scores with the closed genomes of *Syn*AB strains OS-A and OS-B´ ([60]; Supplementary Table 1). The tree was outgroup-rooted with a member of the *Synechococcus* “T1” clade, the sister taxon of *Syn*AB [27, 36, 41]. The six well-supported clades (I-VI) in the phylogeny sorted with respect to collection temperature rather than by geography: strains from cooler environments were basal, with clades from increasingly hotter environments nested within them (Fig. 1). Consequently, some clades are more closely related to *Syn*AB from higher temperatures than they are to more ecologically similar strains. For example, Clade II *Syn*AB, which occupy a similar thermal niche as Clade I (the “B-lineage” of [31]), are the closest relatives of the “A-lineage” Clades III-VI (Fig. 1). Average nucleotide identity (ANI; [56]) between Clade VI strains and metagenome-assembled genome (MAG) WC10meta (Fig. 1), which we assembled from biofilm sampled at the *Syn*AB thermal limit, is >99%; similarly, MAGs reported from different samples along Yellowstone geothermal gradients [61] have >99% ANI to Clade IV strain OS-A, and > 97% ANI to Clade I strain OS-B´, respectively. Consequently, our culture collection well matches the predominant *Syn*AB variation in situ. We conclude that *Syn*AB diversity is the product of a single radiation, with subsequent spread throughout western North America, rather than by convergent colonization of higher temperature habitats within different geographic regions.

### Parapatric gene flow continued following *Syn*AB ecological divergence

By contrast with the strong support for the monophyly of Clades I-VI, metrics of gene tree agreement (gene concordance factors and internode certainty values) with the genome-wide phylogeny were lower both within clades and for deeper splits in the phylogeny (Fig. 1). For two reasons, we believe that gene flow contributes strongly to this observation. Within clades, homologous recombination can occur at high rates [62, 63] and lead to gene tree discordance. We also revealed evidence for the maintenance of continued gene flow between locally co-occurring (i.e. parapatric) clades that have diverged in thermotolerance but partially overlap in distribution. For example, common alternative gene tree topologies grouped Clades II/III and Clades III/V as sister taxa (22% and 15% of gene trees, respectively; Supplementary Fig. 1).

We also uncovered cases of HGT of novel gene content between divergent but parapatric *Syn*AB. For instance, a ~32 kb block containing *nif* genes for nitrogen fixation is present in the outgroup strain Nb3U1 and Clade I, and we consequently infer it to have been present in the *Syn*AB ancestor. By contrast, these genes are missing from all other *Syn*AB, with the exception of several members of Clade IV. A neighbor net analysis suggests that there have been at least two independent HGT events from a Clade I donor (Supplementary Fig. 1), which share similar recombination breakpoints and thereby implicate a site-directed insertion process. We conclude that rare local gene flow between divergent *Syn*AB has continued to shape their evolution and ecology.

### Physiological changes during adaptation to higher temperatures

**Figure 2:**
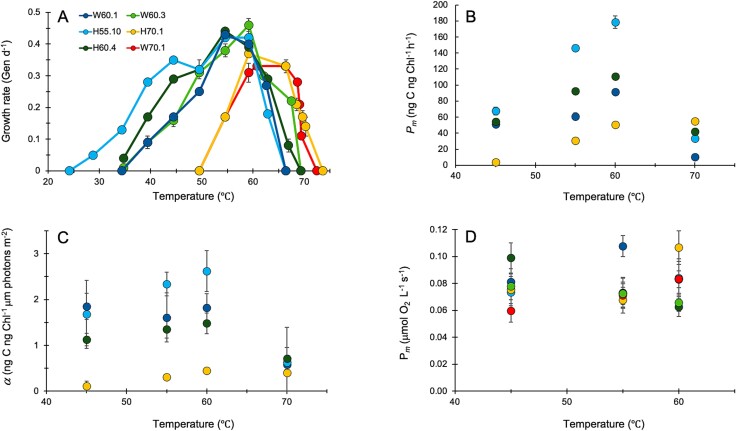
(**A**) Temperature dependence of growth rate for representative *Syn*AB strains from each clade. Error bars are standard errors for triplicate cultures. (**B**) Temperature dependence of maximal carbon fixation rate *P_m_*. Error bars are standard deviations. (**C**) Temperature dependence of the rate constant α (the initial slope of carbon fixation increase with increase in irradiance). Error bars are standard deviations. (**D**) Temperature dependence of maximal oxygen evolution rate *P_m_*. Error bars are standard deviations. For both carbon fixation and oxygen evolution experiments, cells had been grown at optimal temperature prior to assay. Color-coding for B-D as for panel A.

We expected the thermotolerances of representative strains from clade pairs I/II, III/IV and V/VI to respectively resemble each other, based on the environmental temperatures of samples (Fig. 1; Supplementary Table 1), previous investigations of *Syn*AB thermal performance [28–30, 63, 64], and surveys of the distribution of *Syn*AB diversity in situ [31, 61, 65]. This was indeed the case (Fig. 2A). Groups were particularly distinguished by differences in the maximum temperature for growth, *CT_max_*, from below ~65°C for Clade I/II strains, to below 70°C for Clades III/IV, and greater than 70°C for Clades V/VI. These latter strains exhibited both a narrower niche breadth and a lower maximal growth rate, as previously observed [28, 29]. For comparison, optimal growth of outgroup strain Nb3U1 was at ~30°C, with no growth at 55°C (Supplementary Fig. 2).

To investigate the physiological processes impacting *Syn*AB growth, we measured the temperature and light dependence of carbon assimilation and oxygen evolution for representative strains. Carbon fixation by less thermotolerant strains was optimized at 55–60°C, with greatly reduced short-term activity at the lethal temperature of 70°C (Fig. 2B; Supplementary Fig. 3; Supplementary Fig. 4). In the most thermotolerant *Syn*AB, optimal carbon fixation occurred at higher temperatures (Fig. 2B; Supplementary Table 2), albeit with a lower rate constant (α) at all temperatures (Fig. 2C), the lowest maximal rate *P_m_* (Fig. 2B), and negligible activity at 45°C (Fig. 2B). By contrast, *Syn*AB strains exhibited no difference in maximal net photosynthetic oxygen evolution between 45–60°C (Fig. 2D; *F*_5,6_ = 0.95, *P* = 0.51; Supplementary Fig. 5). A significant strain effect for rate constant α (*F*_5,12_ = 16.9, *P* < 0.0001) primarily reflected the comparatively lower compensation point (i.e. the irradiance at which photosynthetic oxygen evolution equals respiratory consumption) for Clade I strain W60.1 (Supplementary Fig. 6). These differences in carbon fixation yet comparable oxygen evolution in common garden thermal and DIC environments point to the reduction in carbon assimilation by the most thermotolerant strain occurring downstream of PSII. This may be the result of the more thermostable but slower carboxylation activity of RuBisCO by the most thermotolerant *Syn*AB [33]. These results also corroborate the previously proposed [66] existence of an alternative, as yet unidentified electron sink(s) (beyond carbon dioxide) for reducing power generated during the light reactions.

### Increased thermotolerance is associated with smaller genomes and the loss of community complexity

**Figure 3:**
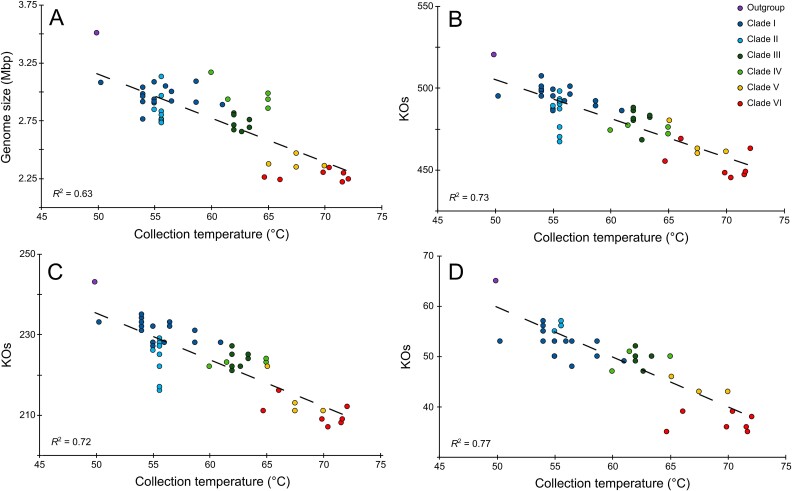
(**A**) *Syn*AB genome size versus sample collection temperature (*N* = 47 genomes with BUSCO coverage >90%). Mean genome size for clade V/VI strains was 80% of that of clade I (mean ± SE: 2.4 ± 0.03 versus 3.0 ± 0.03 Mb). Number of KEGG pathway orthologs (KOs) in each genome versus temperature for (**B**) global metabolic pathways, (**C**) secondary metabolite synthesis, and (**D**) ABC transporters. Dashed lines are least square regression lines.

In bacteria and archaea, genome size is negatively correlated with optimal growth temperature [6]. We also observed a strong negative correlation between genome size and temperature for *Syn*AB (Fig. 3A; *R* = −0.79; *F*_1,45_ = 68.2, *P* < 0.0001 for a PGLS model). Bacterial genome size has also been reported to be positively correlated with cell size [5]. Because DNA can occupy a substantial fraction of the volume of small cells [67], it has been proposed that genome size reduction in thermophiles may be a byproduct of selection for smaller cell size [6] to reduce increased maintenance costs associated with high temperature environments [68–70]. However, more thermotolerant *Syn*AB are not smaller than less tolerant cells (Supplementary Table 3).

We propose that the smaller genomes and reduced metabolic potential of more thermotolerant *Syn*AB instead may be the product of declines in community complexity and environmental heterogeneity along these geothermal gradients. The species richness and metabolic diversity of a community impact the quality, amount, and cycling of resources, the nature of competition, cooperation, and communication among organisms, and the formation and heterogeneity of chemical gradients associated with microbial activity. Environmental stability had been previously rejected as an explanation for genome reduction in thermophiles [6]; however, because detailed environmental information is lacking for most organisms, this was based on an indirect metric of environmental variation (NCBI Entrez Genome Project lifestyle classification). Here, we were able to capitalize on robust information on biogeochemistry and diversity of these communities to address the potential links between environmental variability and genome size. Species diversity, food web complexity, and productivity of these communities are highly temperature-dependent and peaks for the thick, laminated microbial mats found between ~55–61°C [65, 71]. At these lower temperatures, mats exhibit dramatic fluctuations in vertical oxygen and sulfide gradients arising from diurnal patterns of *Syn*AB photosynthesis [71, 72]; they are likewise marked by a complex network of metabolic interactions that are absent at higher temperature. These include the complete anaerobic decomposition of organic matter by acetogenesis and other fermentation pathways [73, 74], sulfate reduction [72], and methanogenesis [75]. Consequently, we expect a more diverse nutrient pool at lower temperatures. This is reflected in *Syn*AB metabolic potential, particularly in the number of genes involved in active transport, secondary metabolite biosynthesis, quorum sensing, and nitrogen metabolism (Fig. 3B-D; Supplementary Fig. 7). For example, less thermotolerant strains possess ABC transporters for sources of organic N (urea, as well as annotated polar amino acid transporter and proline/glycine betaine transporters) that are absent from the genomes of more thermotolerant clades (Fig. 3).

Genome reduction in thermophiles has been proposed to be directly or indirectly favored [6]; however, multiple observations suggest that this may not be the case for *Syn*AB. Selectively favored, streamlined genomes are expected to contain a lower proportion of intergenic DNA [76–78], but the amount of *Syn*AB non-coding DNA was not temperature dependent (Supplementary Table 4). In addition, strains with smaller genomes did not divide faster (Fig. 2), as would be expected if smaller genomes are favored for more rapid reproduction [77]. These results instead suggest the possible role of drift for *Syn*AB genome loss. Although we might expect drift to be weak compared with selection for large *Syn*AB populations, it may potentially have been locally strong during range expansion along the thermal gradient. This could have resulted the fixation of neutral deletions [79–81] that arise from the deletional bias of bacterial genomes [77].

### Limited HGT during *Syn*AB temperature adaptation

**Figure 4:**
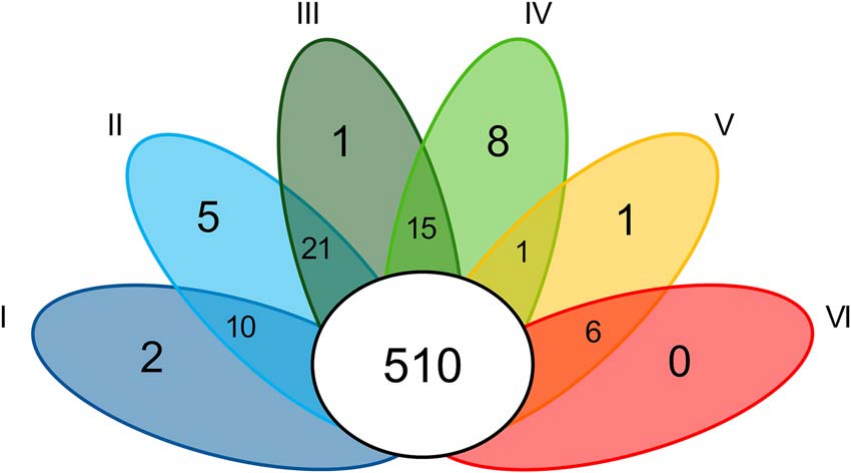
Lotus plot indicating the *Syn*AB core genome (white), unique genes for individual clades, and uniquely shared genes for representative gene pairs.

Thermophilic bacteria can possess a substantial number of genes that have been obtained by HGT [24, 25]. However, the gain of novel gene content by HGT has played a quantitatively much more limited role; comparative genomics revealed only low numbers of uniquely shared gene content within clades or associated with gains in thermotolerance (e.g. six genes for Clades V-VI; Fig. 4). Of these, only a few were identified as strong candidates for HGT based on sequence identity with taxa outside of the *Syn*AB group (Supplementary Table 5). This is similar to the rare occurrence of HGT we observed for other ecological traits between *Syn*AB populations (see above). Examples include genes encoding a vitamin B_12_ (cobalamin)-independent methionine synthase and a YbhB/YbcL family phospholipid-binding protein found in descendants of the Clade III-VI ancestor, as well as molybdenum cofactor synthesis and assimilatory nitrate reductase genes co-acquired by the Clade II–VI ancestor. In the above cases, genes were putatively acquired from other moderately thermophilic, phototrophic community members that are abundant at lower temperatures (e.g. Chloroflexi, the cyanobacterium *Gloeomargarita* [65]; Supplementary Table 5).

The only clear example of a gene acquired by the Clade V/VI ancestor is a copy of *trmH* obtained from a *Thermus* bacterium (Fig. 4A; the *Syn*AB proteins are >88% identical to *T. aquaticus*). TrmH is a methyltransferase that methylates G18 in the D-arm of tRNAs [82]. Post-transcriptional modifications of tRNAs can play an important role in the temperature adaptation of thermophiles [83], and methylation of G18 stabilizes the D-arm, overall tRNA shape, and the tRNA-protein interaction [84–86]. These are type I TrmH enzymes that can modify all tRNA species [87], and TrmH activity is thermally induced in *Thermus thermophilus* [88], implying its role in tRNA stabilization at high temperature. Following HGT, TrmH evolution has largely been constrained by strong purifying selection (e.g. d_N_/d_S_ = 0.04 for the ancestral branch of the *Syn*AB copies), indicating conservation of function. However, GC at third codon positions (GC3) of the *Syn*AB sequences has diverged greatly from *Thermus* (Fig. 4B) to resemble that of *Syn*AB genomes overall. Consequently, *trmH* acquisition may have been a key innovation during *SynAB* colonization of temperatures greater than 70°C.

**Figure 5:**
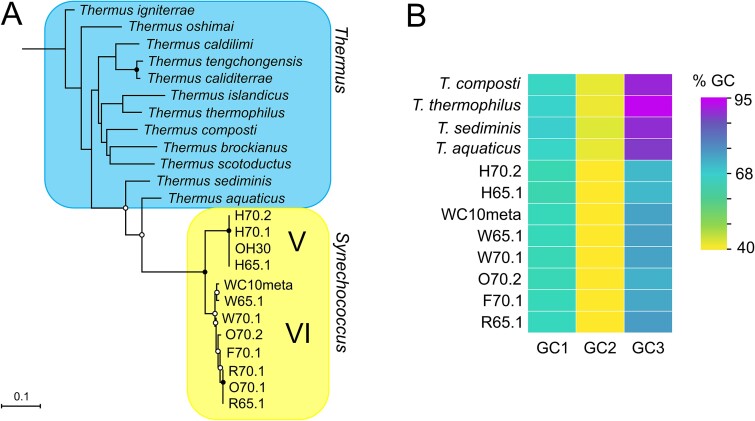
(**A**) *Maximum* likelihood phylogeny of the *trmH* tRNA methyltransferase gene for *Thermus* and *Syn*AB clade V/VI strains reconstructed with the TPM3 + F + G4 model selected by ModelFinder and 1000 bootstrap replicates. The tree is outgroup-rooted with sequence data for *Meiothermus taiwanensis* (GenBank accession CP021130.1). Bootstrap values of 100% are indicated by closed circles, and values greater than 90% are indicated by open circles. Tree scale is in units of nucleotide substitutions per site. (**B**) GC content of *trmH* at first, second and third codon positions.

### Changes in base composition are not associated with increased thermotolerance

A robust correlation between GC content of structural RNAs and optimal growth temperature has been reported [7–9], as has evidence for purine (AG) loading by thermophiles [10–12, 21]. We did not, however, observe a strong relationship between temperature adaptation and either genome-wide GC content, GC3, GC of RNAs, or AG content. Rather, clade I genome-wide GC (95% confidence interval = 58.4,58.6) was slightly lower than that of the other clades, the confidence intervals of which did not differ significantly from each other (*F*_1,32_ = 0.2, *P* = 0.66). This shift in GC did not directly impact temperature adaptation, because clades I and II occupy similar thermal niches (Fig. 1) and have not diverged in thermotolerance (Fig. 2). However, we cannot rule out that higher GC or its correlates, such as codon usage or amino acid composition (see below), may have predisposed the Clade III-VI ancestor to subsequent adaptation to higher temperatures. Similarly, GC3 was lower for Clade I than the rest, but, in addition, the most thermotolerant clades V/VI actually had lower GC3 than Clades II-IV (Supplementary Table 6). Clades V/VI did exhibit slightly higher average GC for RNAs than the others (60% versus 59%; *F*_2,54_ = 17.5, *P* < 0.0001), although Clades I and III were not significantly different than Clade V. Clades also did not differ significantly in AG content (*F*_5,51_ = 0.8, *P* = 0.57).

### Aspartate phobia and the evolution of *Syn*AB amino acid composition

Amino acid composition of *Syn*AB clades did not cluster strictly by thermotolerance in a principal component analysis (Supplementary Fig. 8). Rather, Clade II more closely resembled Clades III/IV than the more phenotypically similar Clade I, which reflects the difference in GC content between Clade I and other *Syn*AB (Fig. 5). Therefore, to address which specific changes in amino acid composition are most strongly associated with increased thermotolerance, we focused on Clades II–VI, which have diverged in CT_max_ (Fig. 2A) but do not exhibit potentially confounding differences in GC content.

**Figure 6:**
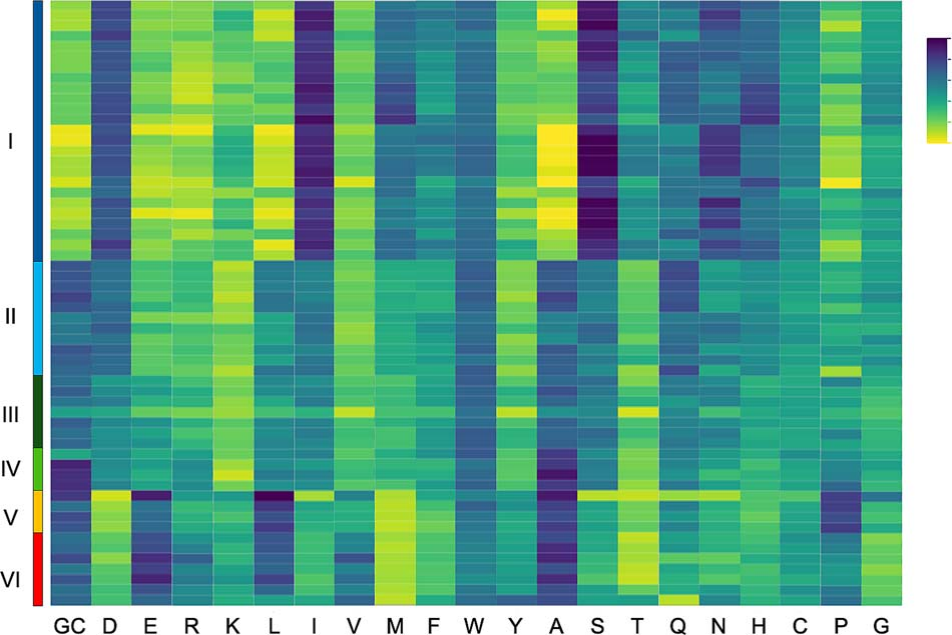
Heat maps of GC content and amino acid compositions for *Syn*AB strains. Percent ranges for heat map gradient: GC (58–61); D (4–4.5); E (6.2–6.7); R (7–7.5); K (2.7–3.2); L (12.3–13); I (4.4–4.9); V (6.8–7.3); M (1.6–1.9); F (3.3–3.6); W (1.6–1.9); Y (2.5–2.8); A (9.7–10.3); S (5.3–5.8); T (4.4–4.9); Q (5.4–5.9); N (2.3–2.6); H (1.8–2.1); C (0.9–1.2); P (6.2–6.5); G (7.6–8.1).

The evolution of *Syn*AB amino acid composition was not as simple as the predicted increases in both charged and bulky hydrophobic residues. The only two amino acids that exhibited a strong association with temperature throughout *Syn*AB diversification were the charged residues aspartate (Asp) and glutamate (Glu) (Fig. 6). The reduction in Asp with increasing environmental temperature represented the greatest relative change in usage of any amino acid (Supplementary Fig. 9; *R*^2^ = 0.73; *F*_1,31_ = 82.1, *P* < 0.0001; slope = −1.6% Asp per 5°C) and explained the most variance among *Syn*AB in a discriminant analysis (Supplementary Fig. 10). This is in accord with the recent proposal that thermophiles exhibit “aspartate phobia” to avoid the temperature-dependent consequences of protein damage at Asp residues [89]. Asp is particularly prone to the spontaneous hydrolysis of peptide bonds [90, 91], which results in the irreversible truncation and impaired function of proteins [92, 93]; Asp isomerization is also a major contributor to protein degradation [94–96]. Rates of Asp modification are highest when the carboxyl adjacent amino acid is glycine [94, 97], and we observed a decrease in the frequency of Asp-Gly in more thermotolerant strains (7.2% in Clade II vs. 6.9% in Clade V/VI) compared with its proteome-wide frequency (7.8 vs. 7.7% Gly, respectively). This reduction in Asp was mirrored by gains in Glu (Supplementary Fig. 9; 1.4% Glu per 5°C; *R*^2^ = 0.80; *F*_1,31_ = 125.7, *P* < 0.0001), principally through GAT-to-GAG transversions, thereby potentially mitigating damage yet maintaining net protein charge.

Our results also revealed widespread remodeling of amino acid composition specifically during the colonization of temperatures greater than 70°C by Clades V/VI. In addition to the replacement of Asp with Glu, we observed increases in Val, Leu, Tyr, and Lys compared with less thermotolerant *Syn*AB, along with decreases in Ile, Met, Phe, Trp, Ser, and Gln (Fig. 5). These idiosyncratic changes at the thermal limit for phototrophy therefore only showed mixed support for the proposed universal set of amino acid usage changes at higher temperatures (IVYWREL; [21]). Although several (Glu, Val, Tyr, Leu) did increase in frequency as predicted, Arg did not change with temperature, and two (Ile, Trp) actually changed in the opposite direction.

Taken together, our combination of lab physiology and genomics approaches sheds new light on the process of *Syn*AB temperature adaptation. Our results provide only limited support for the conventional view regarding the genomic changes that underlie the evolution of thermophily. This emphasizes the importance of complementing the identification of correlational trends identified across a broad phylogenetic scale with focused investigations of close, ecologically divergent relatives for understanding the process of temperature adaptation. It likewise calls for a closer examination of whether phototrophs and chemolithotrophs exhibit systematic differences in temperature adaptation (e.g. regarding the evolution of cell size) that may be related to their metabolic differences and energy availability in the environment. Future work will focus on the identification of the additional electron sink(s) that emerged during *Syn*AB temperature adaptation above 70°C. This greater requirement for reducing power by the most thermotolerant *Syn*AB compared with their relatives could serve other metabolic processes beyond carbon fixation (e.g. detoxification of reactive oxygen species) and/or the increased biosynthesis of reduced macromolecules such as lipids [66]; protons, however, are an unlikely sink, given the absence of an annotated hydrogenase in their genomes. Together with the reduced RuBisCO carboxylation activity of the most thermotolerant clade, the metabolic costs of this sink may act as a constraint on further adaptation to even higher temperatures.

## Supplementary Material

SI_wrae184

Supplementary_Table_S1_wrae184

Supplementary_Table_S4_wrae184

## Data Availability

The sequence datasets generated during and/or analysed during the current study are deposited at NCBI BioProject PRJNA795194, and raw phenotypic datasets are available from the corresponding author on reasonable request.
