# Supplementary material for: Idiosyncratic genome evolution of the thermophilic cyanobacterium Synechococcus at the limits of phototrophy
Source: ISME J. 2024 Sep 25;18(1):wrae184. doi: 10.1093/ismejo/wrae184 (PMC11456837; doi:10.1093/ismejo/wrae184)
Supplement: SI_wrae184 [file si_wrae184.docx]

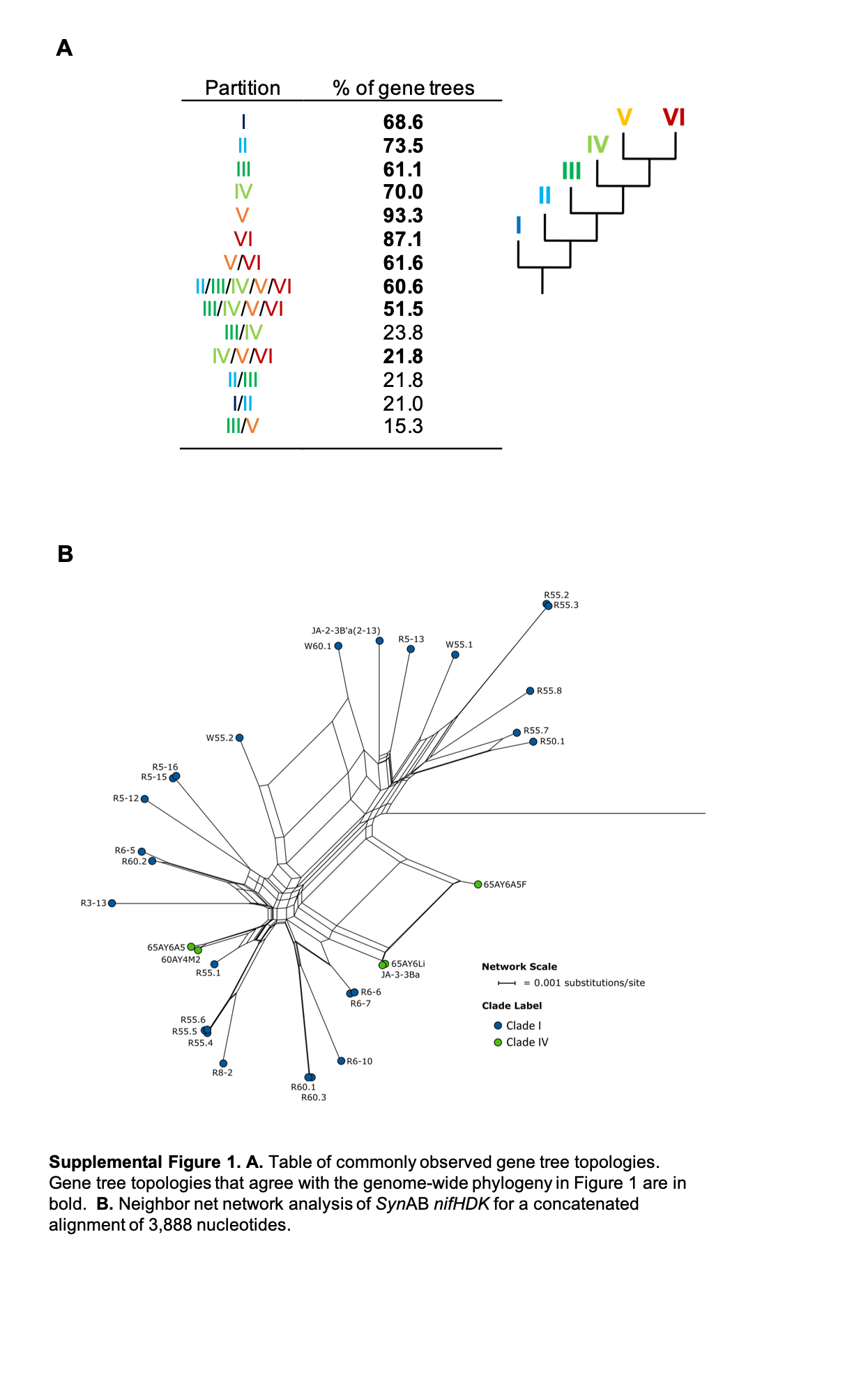


**Supplementary Fig. S1. A.** Table of commonly observed *Syn*AB gene tree topologies. Gene tree topologies that agree with the genome-wide phylogeny in Figure 1 are in bold. **B.** Neighbor net network analysis of *Syn*AB *nifHDK* for a concatenated alignment of 3,888 nucleotides. Remarkably, these HGT events have resulted in the largest syntenic region between the genomes of clade I strain JA-2-3B’a(2-13) and clade IV strain JA-3-3Ab.

**Supplementary Fig. S2.** Temperature dependence of growth rate for outgroup strain *Synechococcus* Nb3U1.


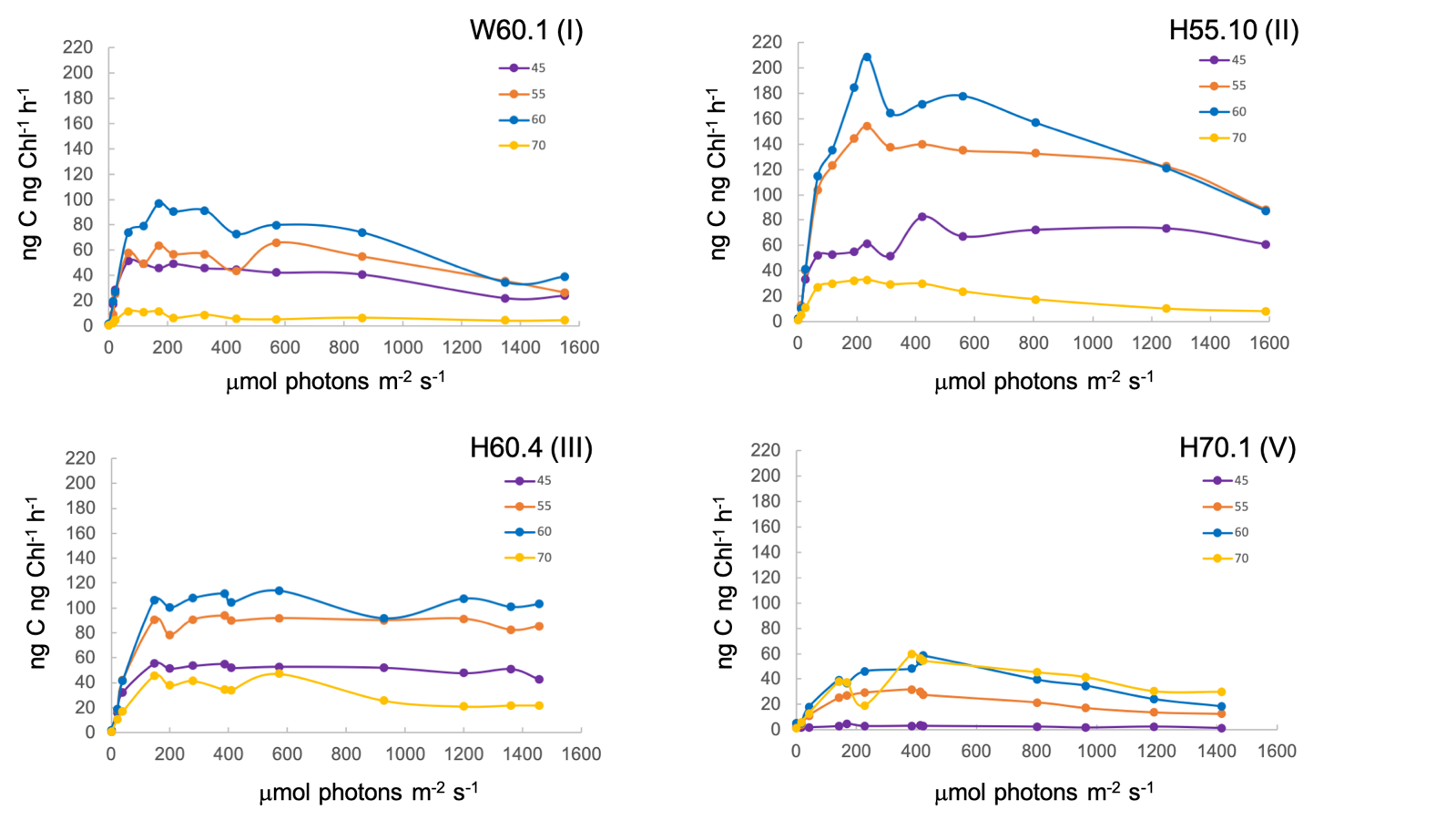


**Supplementary Fig. S3.** Carbon assimilation versus irradiance curves at 45, 55, 60 and 70 °C for representative *Syn*AB strains. Prior to assay, cells had been grown at their optimal maintenance temperature (55 °C: W60.1, H55.10; 60 °C: H60.4; 65 °C: H70.1).


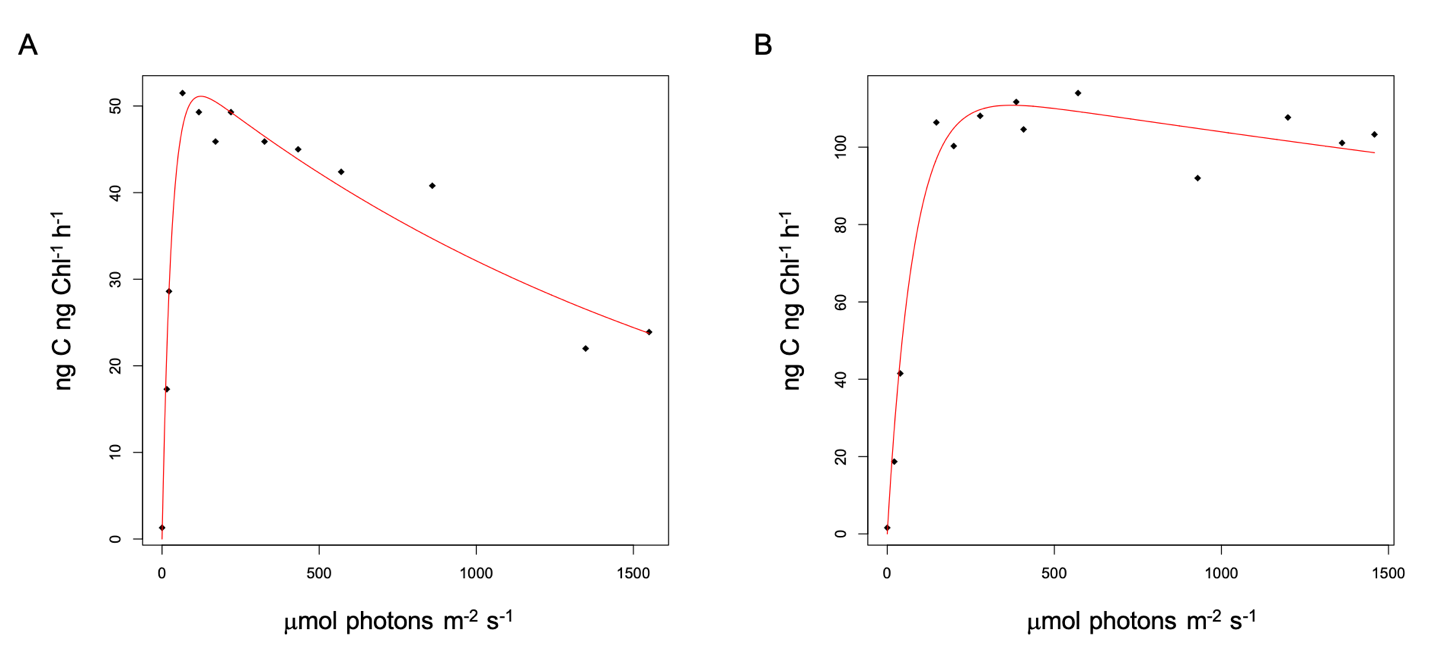


**Supplementary Fig. S4.** Example fits of the Platt model to the *Syn*AB carbon assimilation data, which includes a photoinhibition parameter. **A.** Strain W60.1 at 45 °C. **B.** Strain H60.4 at 60 °C.


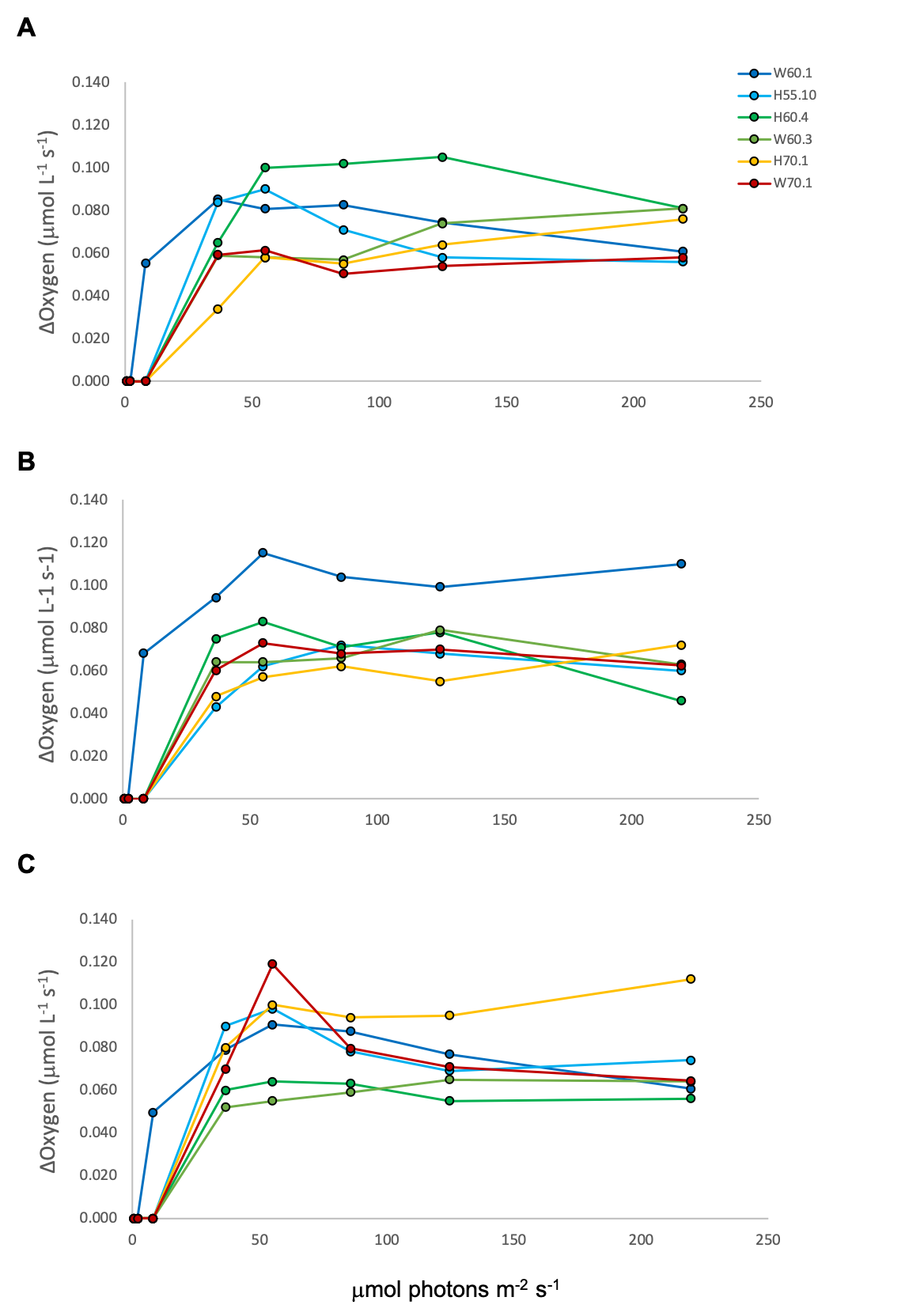


**Supplementary Fig. S5.** Oxygen evolution versus irradiance for representative *Syn*AB strains at (A) 45 °C, (B) 55 °C and (C) 60 °C.


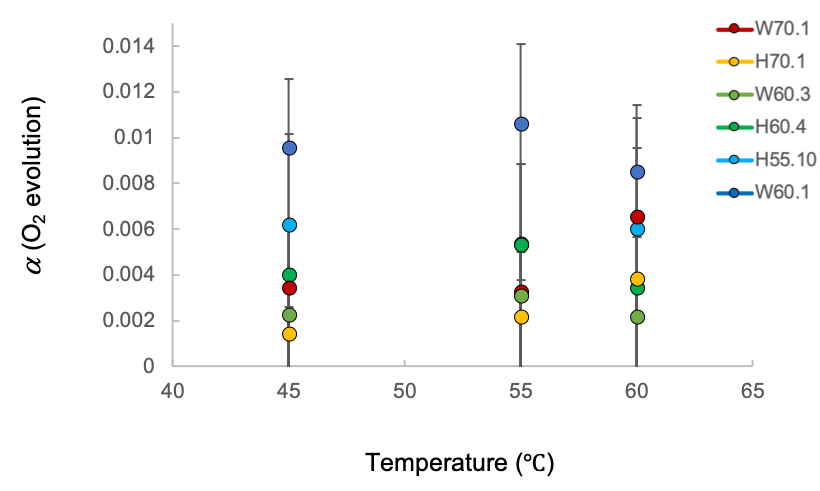


**Supplementary Fig. S6.** Estimates of the rate constant α (i.e., the initial slope of increase in oxygen evolved with increasing irradiance) for Platt model fits to the *Syn*AB oxygen evolution data.


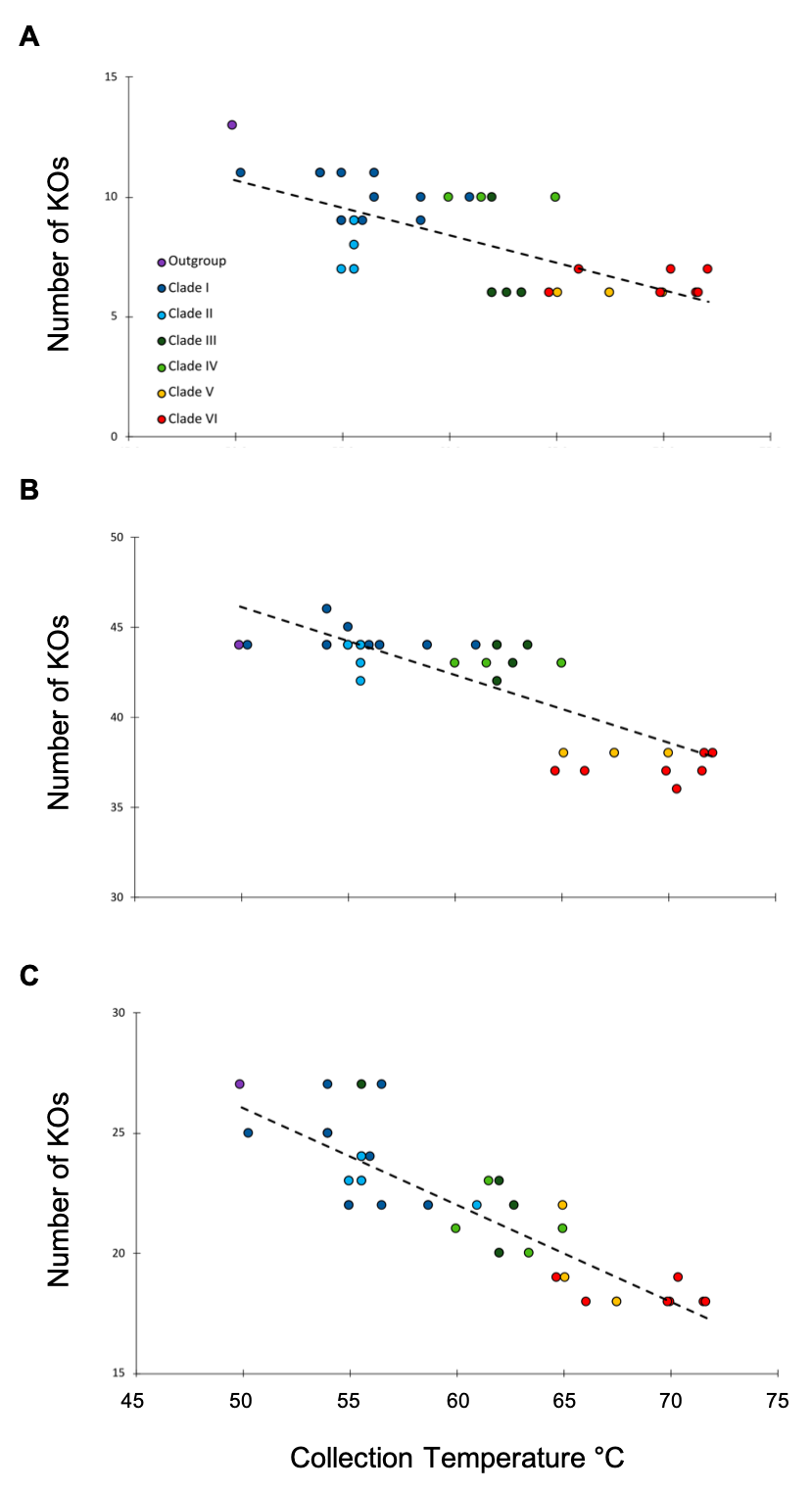


**Supplementary Fig. S7.** Number of KEGG pathway orthologs (KOs) in each genome versus temperature for (**A**) nitrogen metabolism, (**B**) porphyrin metabolism and (**C**) quorum sensing.


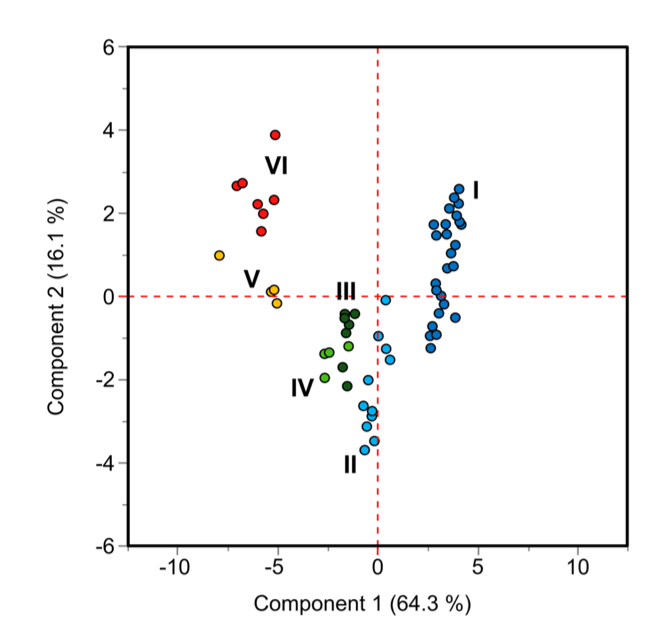


**Supplementary Fig. S8.** Principal component analysis plot for *Syn*AB amino acid composition.


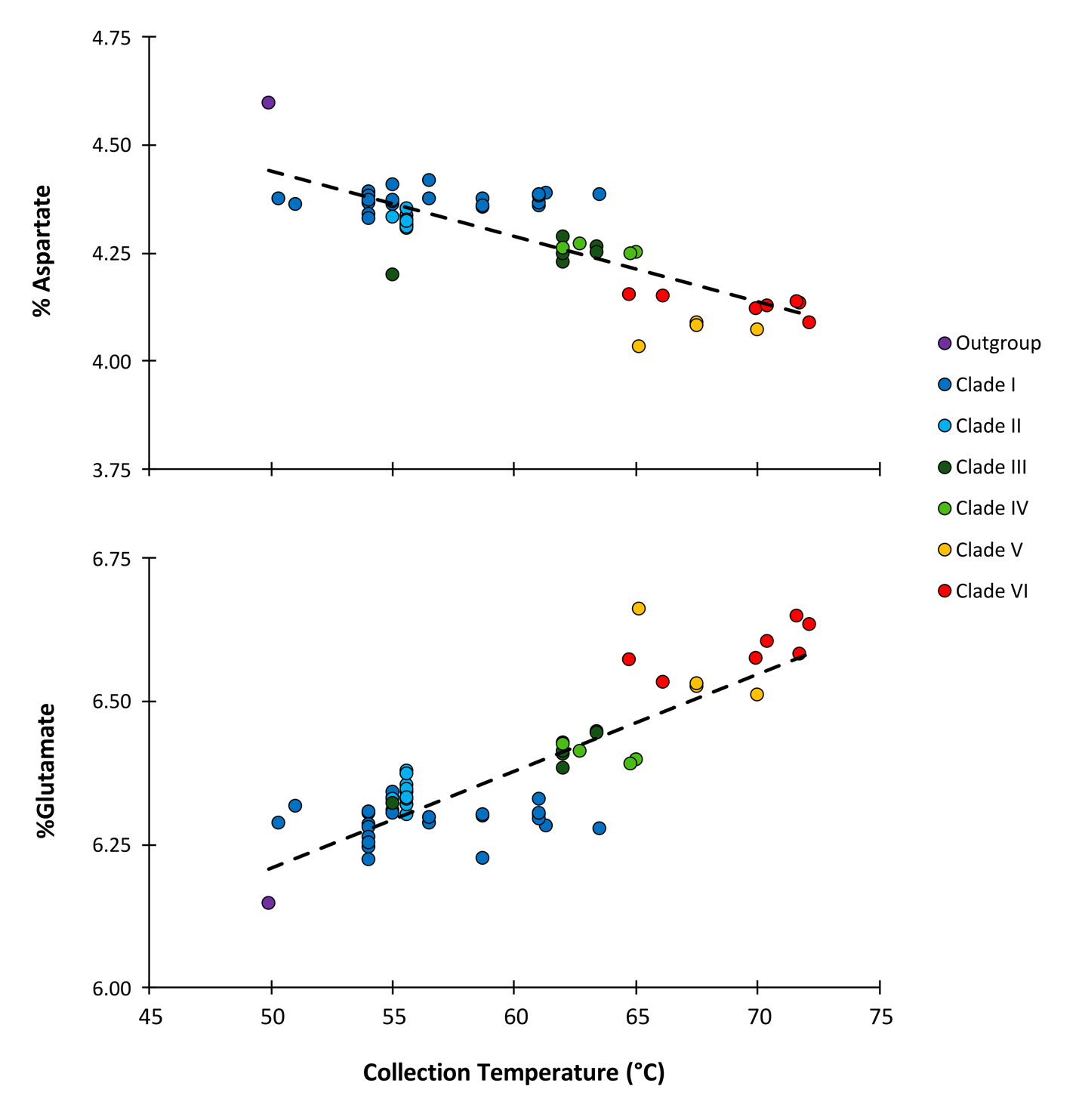


**Supplementary Fig. S9.** Changes in aspartate and glutamate content during *Syn*AB divergence.


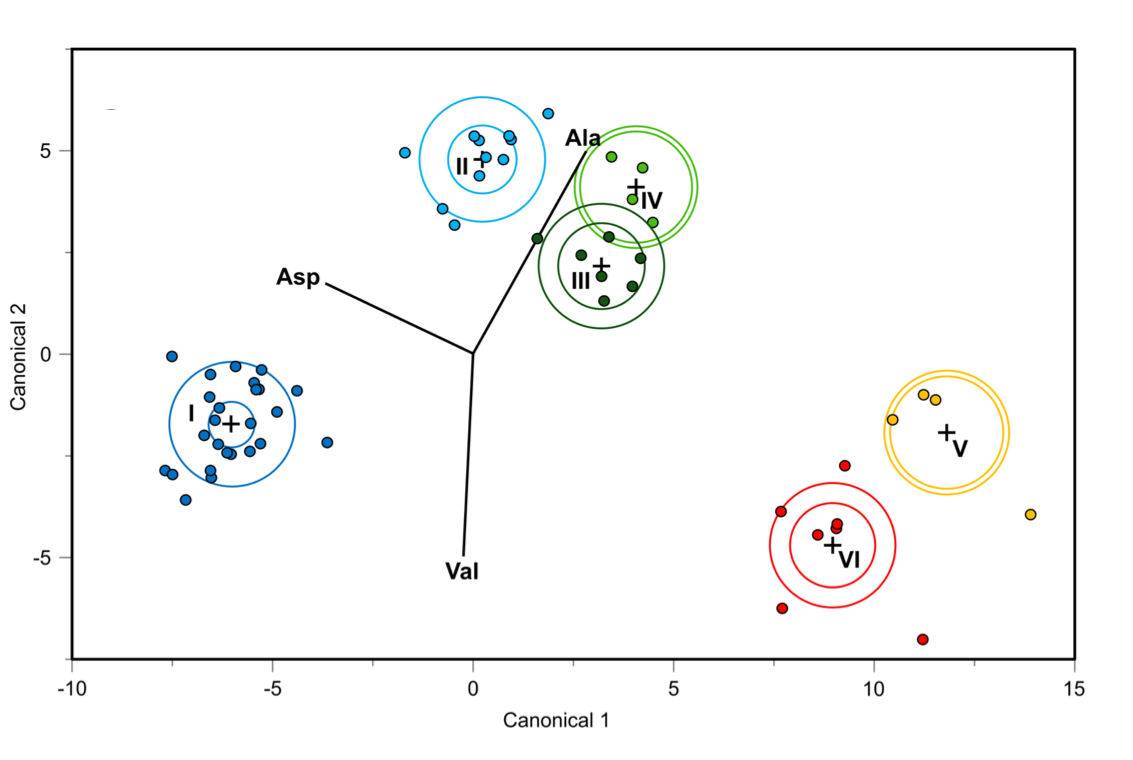


**Supplementary Fig. S10.** Discriminant analysis for *Syn*AB amino acid composition. The analysis could correctly assign all strains to the proper clade by stepwise addition of only three variables with the greatest *F* ratios in ANCOVA tests: % aspartate (*F* = 296.2, *P* = 0), % alanine (*F* = 59.0, *P* = 0), and % valine (*F* = 35.8, *P* = 0).

| **Supplementary table S2.** Parameter estimates for Platt model fits to carbon fixation data. | | | | | |
| --- | --- | --- | --- | --- | --- |
|  | | | | | |
|  |  |  |  |  |  |
| Strain | Temperature (°C) | α (SD)*^a^* | *P_m_* (SD)*^b^* | *I_m_* (SD)*^c^* | β (SD)*^d^* |
|  |  |  |  |  |  |
|  |  |  |  |  |  |
| W60.1 (I) | 45 | 1.85 (0.283) | 51.0 (1.87) | 126 (15.5) | 0.03 (0.006) |
|  | 55 | 1.61 (0.538) | 60.9 (4.39) | 178 (44.2) | 0.03 (0.014) |
|  | 60 | 1.82 (0.310) | 91.5 (3.73) | 201 (25.1) | 0.07 (0.015) |
|  | 70 | 0.58 (0.812) | 10.2 (1.19) | 108 (37.8) | 0.01 (0.011) |
|  |  |  |  |  |  |
| H55.10 (II) | 45 | 1.68 (0.743) | 67.7 (4.94) | 280 (107.7) | 0.01 (0.009) |
|  | 55 | 2.34 (0.263) | 146.4 (4.18) | 273 (23.9) | 0.05 (0.012) |
|  | 60 | 2.62 (0.445) | 178.6 (7.72) | 282 (35.8) | 0.08 (0.022) |
|  | 70 | 0.63 (0.057) | 33.2 (0.79) | 184 (12.2) | 0.05 (0.007) |
|  |  |  |  |  |  |
| H60.4 (III) | 45 | 1.13 (0.137) | 54.6 (1.41) | 253 (25.8) | 0.01 (0.003) |
|  | 55 | 1.35 (0.193) | 92.4 (2.64) | 381 (54.5) | 0.01 (0.005) |
|  | 60 | 1.48 (0.225) | 110.7 (3.62) | 390 (56.4) | 0.01 (0.008) |
|  | 70 | 0.71 (0.239) | 41.9 (2.70) | 236 (50.7) | 0.04 (0.013) |
|  |  |  |  |  |  |
| H70.1 (V) | 45 | 0.11 (0.111) | 3.9 (0.56) | 212 (109.8) | 0.02 (0.021) |
|  | 55 | 0.31 (0.034) | 30.5 (0.91) | 309 (23.4) | 0.05 (0.013) |
|  | 60 | 0.45 (0.060) | 50.8 (1.97) | 360 (32.3) | 0.08 (0.021) |
|  | 70 | 0.40 (0.034) | 54.7 (1.49) | 426 (24.5) | 0.08 (0.019) |
|  |  |  |  |  |  |

*^a^* Photochemical rate constant (initial slope of P versus I curve)

*^b^* Maximal estimated rate of carbon fixation

*^c^* Irradiance of maximal rate of carbon fixation

*^d^* Photoinhibition parameter

| **Supplementary table S3.** Cell size estimates*^a^* for representative *Syn*AB cells. | | | | | |
| --- | --- | --- | --- | --- | --- |
|  |  |  |  |  |  |
| Strain | Clade | Mean length*^b^* (μm) | SD | Mean width*^c^* (μm) | SD |
|  |  |  |  |  |  |
| R50.1 | I | 4.064 | 0.753 | 1.383 | 0.171 |
| H55.3 | II | 4.338 | 1.070 | 1.458 | 0.173 |
| H60.1 | III | 6.481 | 1.559 | 2.217 | 0.213 |
| H70.1 | V | 5.630 | 0.971 | 1.683 | 0.294 |
| W70.1 | VI | 5.156 | 0.785 | 1.400 | 0.170 |
|  |  |  |  |  |  |

*^a^* Cell dimensions were estimated with ImageJ for 30 cells of each strain.

*^b^ R*^2^ for the linear regression of mean length on environmental temperature was 0.37 (*P* = 0.28 for an *F* test).

*^c^ R*^2^ for the linear regression of mean length on environmental temperature was 0.03 (*P* = 0.79 for an *F* test)

| **Supplementary table S5.** Horizontal gene transfer candidates associated with temperature adaptation.*^a^* | | | |
| --- | --- | --- | --- |
|  |  |  |  |
| Gene | W70.1 gene number | Putative donor | *Syn*AB distribution |
|  |  |  |  |
|  |  |  |  |
| Assimilatory nitrate reductase (*narB*) | 583 | Cyanobacterium | II-VI*^b^* |
| Nitrate reductase-associated protein | 582 | Cyanobacterium | II-VI |
| Molybdopterin molybdenumtransferase (*moeA*) | 581 | *Gloeomargarita* | II-VI*^c^* |
| GTP 3',8-cyclase (*moaA*) | 580 | *Gloeomargarita* | II-VI |
| Cyclic pyranopterin monophosphate synthase (*moaC*) | 579 | *Gloeomargarita* | II-VI |
| Molybdenum cofactor biosynthesis protein (*moaD*) | 578 | *Gloeomargarita* | II-VI |
| Molybdopterin synthase catalytic subunit (*moaE*) | 577 | *Gloeomargarita* | II-VI |
|  |  |  |  |
| Vitamin-B_12_-independent methionine synthase (*metE*) | 1304 | *Gloeomargarita* | III-VI*^d^* |
|  |  |  |  |
| YbhB/YbcL family phospholipid-binding protein | 1833 | Chloroflexi | III-VI |
|  |  |  |  |
| **tRNA (guanosine(18)-2'-O)-methyltransferase** (*trmH*) | 267 | *Thermus* | V-VI |
|  |  |  |  |

*^a^* All candidates were identified as a taxon other than *Syn*AB by Kraken and not present in the outgroup *Synechococcus* strain Nb3U1 genome based on local blastx analysis. Putative donors were identified based on BLAST hits to the NCBI nr database.

*^b^* Clade I has retained the ancestral copy of *narB*, which is adjacent to nitrite reductase (*nirA*) and nitrate transporter genes.

*^c^* In these clades, ancestral copies of these Moco biosynthesis genes have been lost, but the ancestral copy of *moeB* has been retained.

*^d^* The ancestral copy of Vitamin-B_12_-dependent methionine synthase *metH* has been retained in all *Syn*AB except for Clade VI strains.

| **Supplementary table S6.** % GC3 for *Syn*AB genomes. | | |
| --- | --- | --- |
|  |  |  |
| Clade | % GC3 | SE |
|  |  |  |
| I | 69.2 | 0.07 |
| II | 72.5 | 0.10 |
| III | 72.1 | 0.13 |
| IV | 73.5 | 0.17 |
| V | 71.0 | 0.17 |
| VI | 71.0 | 0.13 |
|  |  |  |
